# Heart rate monitoring on the stroke unit. What does heart beat tell about prognosis? An observational study

**DOI:** 10.1186/1471-2377-11-47

**Published:** 2011-04-27

**Authors:** Martin A Ritter, Anne Rohde, Peter U Heuschmann, Rainer Dziewas, Jörg Stypmann, Darius G Nabavi, Bernd E Ringelstein

**Affiliations:** 1 Department of Neurology, University of Münster, Münster, Germany; 2 Center for Stroke Research Berlin, Charité University Medicine Berlin, Berlin, Germany; 3 Department of Cardiology, University of Münster, Münster, Germany; 4 Department of Neurology, Vivantes Klinikum Neukölln, Berlin, Germany

## Abstract

**Background:**

Guidelines recommend maintaining the heart rate (HR) of acute stroke patients within physiological limits; data on the frequency and predictors of significant deviations from these limits are scarce.

**Methods:**

Demographical data, stroke risk factors, NIH stroke scale score, lesion size and location, and ECG parameters were prospectively assessed in 256 patients with ischemic stroke. Patients were continuously monitored for at least 24 hours on a certified stroke unit. Tachycardia (HR ≥120 bpm) and bradycardia (HR <45 bpm) and cardiac rhythm (sinus rhythm or atrial fibrillation) were documented. We investigated the influence of risk factors on HR disturbances and their respective influence on dependence (modified Rankin Scale ≥ 3 after three months) and mortality.

**Results:**

HR ≥120 bpm occurred in 39 patients (15%). Stroke severity (larger lesion size/higher NIHSS-score on admission), atrial fibrillation and HR on admission predicted its occurrence. HR <45 bpm occurred in 12 patients (5%) and was predicted by lower HR on admission. Neither HR ≥120 nor HR <45 bpm independently predicted poor outcome at three moths. Stroke location had no effect on the occurrence of HR violations. Clinical severity and age remained the only consistent predictors of poor outcome.

**Conclusions:**

Significant tachycardia and bradycardia are frequent phenomena in acute stroke; however they do not independently predict clinical course or outcome. Continuous monitoring allows detecting rhythm disturbances in stroke patients and allows deciding whether urgent medical treatment is necessary.

## Background

Cardiac complications are frequent after acute stroke and, vice versa, cardiac disease is a frequent cause of stroke. About 30% of all strokes are classified as cardioembolic with atrial fibrillation (AF) being the single most important risk factor [1]. The prominent cardiac complications are known since a long time and include myocardial infarctions (MI), tachycardia, and bradycardia [2]. Heart rate (HR) monitoring and maintenance of a normal HR is an essential part of stroke unit treatment [3,4]. However, in contrast to blood pressure, no definition of what is a normal HR is given, and questions arise from this recommendation: What to do when HR leave "physiological limits"? Is active treatment necessary in any case? How frequent are significant tachycardias or bradycardias in an unselected cohort of stroke patients, and at which time point after stroke do they occur? Are there predictors of tachy- or bradycardia? And finally, are tachy- or bradycardia predictive of poor outcome? Data on all these questions is scarce.

## Methods

We included 256 consecutive patients with acute ischemic stroke (< 48 h since symptom onset) admitted to our stroke unit. Patients were classified according to the TOAST criteria with respect of the presumptive cause of stroke [5]. Patients with transient neurological deficits were included in case imaging identified an acute ischemic lesion consistent with the clinical presentation. In total, 365 patients were screened for eligibility. Patients with sinus thrombosis (n = 6), primary or secondary intracerebral haemorrhage (ICH, n = 41), and patients with subarachnoid haemorrhage (n = 5) were excluded. We excluded 17 patients, who were intubated during the admission process for coma or respiratory failure (n = 5) or for emergency intervention demanding anaesthesia (intra-arterial thrombolysis/mechanical thrombectomy, n = 12). We also excluded patients who had to be intubated within 24 hours (n = 6) and before any endpoint occurred, because in all these cases the course of HR was biased by mechanisms other than the natural course, e.g. adrenergic vasopressors to maintain a sufficient blood pressure in sedated patients. We excluded patients who spent <24 hours on the unit (n = 34) and who were referred in stable conditions, as for those only incomplete data sets were available.

The local ethics committee (Ethikkommission der Ärztekammer Westfalen-Lippe) approved data collection and a telephone interview after three months. Follow-up data were provided by the "Qualitätssicherungsprojekt Schlaganfall Nordwestdeutschland" (Quality control project "Stroke" North-Western Germany).

All patients or their proxies gave informed consent to the protocol at the start of the monitoring period.

Demographical data (age, sex, past medical history, time from symptom onset) were assessed. History of chronic heart failure (CHF), coronary heart disease (CHD), AF, and other rhythm disturbances were assessed. Vital signs (bood pressure, oxygen saturation) and laboratory parameters (blood glucose, cholesterol, Troponin I, Creatinine, C-reactive protein) were assessed on admission.

Stroke severity on admission was determined using the National Institute of Health Stroke Scale (NIHSS) [6]. We also noted whether patients received thrombolytic therapy. Time from symptom onset was recorded in full hours.

All patients received cerebral imaging (95% CCT, 69% MRI) to determine lesion size and location. Lesion size was categorized as follows: "1" No infarction visible on scan but clinical symptoms persisting 24 hours or more. "2" infarction <1.5 cm diameter, "3" size up to 1/3 of MCA territory or 1.5-5 cm diameter. "4" lesion 1/3-2/3 MCA territory or >5 cm diameter but no space occupying effect. "5" >2/3 MCA territory or >5 cm diameter plus space occupying effect [7].

Location of the infarction was also assessed on cerebral imaging and classified as "left" and "right" for the left and right anterior circulation territory, "posterior" for lesions only in the vertebro-basilar territory, "multiple" for multiple lesions in more than one pre-defined region and "undetermined" in case no definite new lesion was seen.

### Assessment of heart rate and rhythm

An ECG was recorded on admission and evaluated concerning HR, type of rhythm, configuration and duration of PQ-time, QRS-complex and ST-segment.

Rhythm was described as sinus rhythm or AF. Sinus arryhthmia and atrial and ventricular premature beats were classified as sinus rhythm.

PQ-time was defined as normal or AV-block. Length of the QRS-complex and presence of a pathological Q-wave was noted. The ST-segment was evaluated in patients without branch blocks. Signs of left ventricular hypertrophy (LVH) according to the modified Cornell criteria, signs of chronic strain, and markers of old and acute myocardial infarction were noted [8]. QTc-time was calculated according to the Bazett's-formula.

Acute coronary syndromes (ACS) were diagnosed according to clinical and laboratory investigations. Control ECGs were performed if indicated [9,10].

### Monitoring

Patients were connected to an automated multimodal monitoring system (Siemens SC6000 or SC9000XL, Siemens, Erlangen, Germany) using a three-lead ECG-tracing method routinely displaying lead II of the Einthoven leads. HR was continuously recorded. "Tachycardia" was defined as HR ≥120 beats per minute (bpm), "bradycardia" was defined as HR <45 bpm. These thresholds were arbitrarily chosen prior to the study after consented decision of all authors as "HR that is significantly abnormal and is likely to demand active medical treatment". The textbook definition of tachycardia (≥100 bpm) and bradycardia (<60 bpm) were assessed for comparison reasons. Only the first 24 hours were analysed concerning HR threshold violations to determine the predictive value obtainable in patients monitored for at least this period. The exact timing of the first occurrence of brady- or tachycardia was noted. All patients received echocardiography to assess left ventricular function as an important predictor of outcome in patients with AF [11]. Left ventricular function was graded as normal (ejection fraction, EF >55%), moderately impaired (EF 35-55%) and severely impaired (EF < 35%). Due to the small number of patients with severely impaired EF, data were dichotomised for analysis comparing patients with EF > 55% vs. patients with lower EF. Infectious complications were assessed as described elsewhere [7].

### Treatment of tachycardia/Bradycadia

Active medical treatment was left to the discretion of the treating physician, but was considered in case of "significant" tachycardia or bradycardia as defined above. Furthermore, any tachycardia or bradycardia with clinical symptoms (dizziness, palpitation, pain, systolic BP < 100 mmHg) was considered significant irrespective of the registered heart rate. In case medical treatment was necessary, this was defined as the end point heart rhythm disturbance, HRD. The occurrence of HRDs was recorded for the complete stay in the hospital even beyond 24 hours.

Tachycardic HRD was treated depending on the type of rhythm disturbance. The most frequent type of tachyarrhythmia secondary to AF was treated by metoprolol i.v. (5 mg iv. shot or via continuous infusion, 50 mg/50 ml, at a rate of 2-8 ml/h). Alternatively verapamil i.v. was given (5-10 mg i.v. shot, or continuous infusion 50 mg/50 ml, at a rate of 2-8 ml/h). In addition, digoxin preparations were given iv (0.25 mg, tid for 3 days, thereafter according to serum levels). Pharmakologic cardioversion was performed by amiodarone (900 mg/d iv for 5 days followed by 200 mg/d) or electric cardioversion, the latter especially in cases of instable blood pressure. Bradycardia was only treated, if associated with clinical symptoms or in case of RR intervals >5 s. The first action included atropine (0.1 mg i.v.), attachable external pacer electrodes were placed in selected cases. Indication for permanent pacemaker placement was discussed with the cardiologist. Other rhythm disturbances were treated according to established guidelines for advanced cardiac life support.

### Follow-up

Patients were followed-up clinically throughout their hospital stay and after discharge by validated standardized telephone interviews 3 months after symptom onset to determine clinical outcome [6]. Outcome was determined using the modified Rankin scale (mRS). Dependency was defined as mRS ≥3. Mortality was noted separately.

### Statistics

Patients with tachycardia or bradycardia were compared with patients without events. Single factor analysis was performed using Fisher's exact test for dichotomized items and Chi-square for trend analysis for ordinal items. Mann-Whitney-U tests were performed for continuous variables. HR values obtained at various time points during the first 24 hours were compared using the Wilcoxon Test for matched pairs.

Multiple logistic regression analysis was used to determine independent predictors of HRDs, infectious complications and bad outcome which always included the HR threshold with the models. Lesion size (ordinal 0-5) but not NIHSS on admission was included in these models. Frequency and timing of tachycardia and bradycardia within the first 24 hours were graphically displayed cumulative percentages. Patients

## Results

Basic demographical data of the study population and of the patients who had to be excluded, because they did not complete the 24 study period as described above are given in table 1.

**Table 1:** Basic demographical data of study population and excluded patients

	Study population (n = 256)	Excluded stroke patinets (n = 40)	p
**Male**	135 (53)	25 (63)	n.s.
**Age [years]**	70 (61-76,5)	67 (55,25-73,25)	n.s.
**time to admission [h]**	2,3 (1,1-6,0)	3,00 (2,3-11,0)	0,01
**NIHSS**	5 (2-9)	1 (1-3,25)	0.0001

**Lesion size**	2 (2-3)	2 (1-3)	0.05
**Infarction site**: Right	73 (29)	11 (28)	n.s.
left	98 (38)	10 (25)	
posterior	47 (18)	11 (28)	
multiple	4 (10)	1 (3)	
none	28 (11)	7 (18)	

**aHT**	193 (75)	27 (68)	n.s.
**COPD**	16 (6)	3 (8)	n.s.
**DM**	60 (23)	7 (18)	n.s.
**CHOL**	53 (21)	8 (20)	n.s.
**STROKE**	54 (21)	12 (30)	n.s.
**MI**	15 (6)	4 (10)	n.s.
**CHF**	24 (9)	4 (10)	n.s.
**CAD**	43 (17)	11 (28)	n.s.
**AF**	62 (24)	6 (15)	n.s.

**1. BP syst [mmHg]**	160 (140-180)	156 (146-170)	n.s.
**1. BP diast [mmHg]**	88 (78,75-99)	89,5 (81,5-92,75)	n.s.
**SaO2 [%]**	97 (95-98)	98,5 (97-100)	n.s.
**GCS**	15 (15-15)	15 (14-15)	n.s.
**HR [bpm]**	80 (68-90)	72,5 (64-83,5)	0.04

**thrombolysis**	52 (20)	4 (10)	n.s.

aHT - arterial hypertension, COPD - chronic obstructive pulmonary disease, CHF - chronic heart failure, CHD - coronary heart disease, AF - atrial fibrillation, BP - blood pressure, HR - heart rate, GCS - Glasgow Coma Scale. Ordinal and continuous variables are median (IQR), dichotomous variables are n(%).

Patients who had to be excluded from the final analysis had longer times to admission (p = 0.01), had lower heart rates on admission (0.04) and had lower NIHSS scores on admission (p < 0.001). However, those patients that had to be excluded because of clinical deterioration and subsequent intubation had significantly higher NIHSS scores on admission (15, 7.5-15.5 vs. 5, 2-9; p < 0.01).There were no significant differences in other demographical parameters.

Detailed data of single factor analysis results of the study population are given in table 2.

**Table 2:** Basic data and single factor analysis for tachy- and bradycardia

	<45 bpm (n = 12)	≥45 (n = 244)	p	≥**120 (n = 39)**	<120 (n = 217)	p	≥**100 (n = 87)**	<100 (n = 169)	p	≥60 (n = 142)	<60 (n = 114)	p
**Male**	8 (67)	127 (52)	n.s.	23 (59)	112 (52)	n.s.	45 (52)	90 (53)	n.s.	67 (47)	68 (60)	0.06
**Age [years]**	72,5 (64,5-79)	70 (61-79)	n.s.	74 (64,5-78)	70 (59-78)	0.09	69 (62-77)	71 (59-79)	n.s.	70 (61-78)	72 (62-78)	n.s.
**time to admission [h]**	1,8 (1,1-3,2)	2,3 (1,2-3,2)	n.s.	1,8 (1,1-3,4)	2,5 (1,2-6,8)	n.s.	2 (1,1-5,6)	2,5 (1,2-7,1)	n.s.	2 (1,1-5,8)	2,6 (1,2-7)	n.s.
**NIHSS**	3 (2-8)	5 (2-9)	n.s.	7 (3,5-12,5)	4 (2-8)	0.003	6 (3-11,5)	4 (2-7)	0.0004	5,5 (2-9)	4 (2-8)	n.s.

**infarction site**: right	4 (33)	69 (28)	n.s.	11 (28)	62 (29)	n.s.	22 (25)	51 (30)	0.06	42 (30)	31 (27)	n.s.
left	5 (33)	94 (38)		20 (51)	78 (36)		42 (48)	56 (33)		57 (40)	41 (36)	
posterior	3 (25)	44(18)		5 (13)	42 (19)		16 (18)	31 (18)		24 (17)	23 (20)	
multiple	0	10 (4)		1 (3)	9 (4)		3 (3)	7 (4)		7 (5)	3 (3)	
none	1 (8)	27 (11)		2 (5)	27 (12)		4 (5)	25 (15)		12 (8)	16 (14)	

**Lesion size**	2,5 (2-3)	2 (2-3)	n.s.	3 (2-4)	2 (2-3)	<0.0001	3 (2-3)	2 (2-3)	0.01	2 (2-3)	2 (2-3)	n.s.
**aHT**	10 (83)	183 (75)	n.s.	33 (85)	160 (74)	n.s.	72 (83)	121 (72)	0.06	111 (78)	82 (72)	n.s.
**COPD**	0	16 (7)	n.s.	3 (8)	13 (6)	n.s.	6 (7)	10 (6)	n.s.	11 (8)	5 (4)	n.s.
**DM**	3 (25)	44 (18)	n.s.	5 (13)	55 (25)	n.s.	16 (18)	44 (26)	n.s.	36 (25)	24 (21)	n.s.
**CHOL**	4 (33)	49 (20)	n.s.	4 (10)	49 (23)	0.08	14 (16)	39 (23)	n.s.	24 (17)	29 (25)	n.s.
**STROKE**	5 (43)	49 (20)	0.13	8 (21)	46 (21)	n.s.	17 (20)	37 (22)	n.s.	28 (20)	26 (23)	n.s.
**MI**	1 (8)	14 (6)	n.s.	2 (5)	13 (6)	n.s.	3 (3)	12 (7)	n.s.	6 (4)	9 (8)	n.s.
**CHF**	2 (17)	22 (9)	n.s.	1 (3)	23 (11)	n.s.	5 (6)	19 (11)	n.s.	13 (9)	11 (10)	n.s.
**CAD**	3 (25)	40 (16)	n.s.	7 (18)	36 (17)	n.s.	15 (17)	28 (17)	n.s.	22 (15)	21 (18)	n.s.
**AF**	5 (42)	57 (23)	n.s.	23 (59)	39 (18)	<0.0001 (OR 6.6; 3.2-13.5)	36 (41)	26 (15)	<0.0001 (OR 3.9; 2.1-7.0)	38 (27)	24 (21)	n.s.

**Vital signs/admission**												
1. BP syst [mmHg]	167 (140-184)	160 (140-180)	n.s.	163 (140-180)	160 (140-180)	n.s.	160 (140-181)	160 (140-180)	n.s.	160 (140-180)	164 (140-180)	n.s.
1. BP diast [mmHg]	80 (73-97)	88,5 (80-99)	n.s.	90 (80-100)	85 (75-96)	0.04	90 (80-100)	85 (75-95)	0.04	90 (80-100)	83 (76-95)	0.07
SaO2 [%]	98,5 (97-99)	97 (95-97)	n.s.	97 (95-98)	97 (95-98)	n.s.	97 (95-98)	97 (96-98)	n.s.	97 (95-98)	98 (96-98)	n.s.
GCS	15 (13-15)	15 (15-15)	n.s.	15 (12,5-15)	15 (15-15)	n.s.	15 (15-15)	15 (13-15)	n.s.	15 (14-15)	15 (15-15)	n.s.
HR [bpm]	54,5 (50-68)	80 (70-90)	<0.0001	104 (88,5-121)	77 (65-85)	<0.0001	92 (80-108)	74 (65-80)	<0.0001	85 (77-97)	70 (61-80)	<0.0001

**thrombolysis**	2 (17)	50 (20)	n.s.	10 (26)	42 (19)	n.s.	21 (24)	31 (18)	n.s.	32 (23)	20 (18)	n.s.

**Laboratory values**												
TC [mg/dl]	154 (137-180)	182 (159-213)	n.s.	168 (153-192)	182 (157-214)	n.s.	183 (158-212)	180 (156-212)	n.s.	182 (158-212)	177 (155-211)	n.s.
HbA1c [%]	5,8 (5,6-6,2)	5,8 (5,6-6,2)	n.s.	5,9 (5,6-6,1)	5,8 (5,6-6,2)	n.s.	5,9 (5,6-6,1)	5,8 (5,5-6,2)	n.s.	5,9 (5,6-6,3)	5,8 (5,6-6,1)	n.s.
Glc. [mg/dl]	125,5 (105-164,5)	111,5 (98-135)	n.s.	117 (105-141)	111 (97-13)	n.s.	120 (106-149)	107 (95-133)	0.004	116 (98-146)	108 (96-132)	
TropI [ng/ml]	0,03 (0,01-0,06)	0,02 (0,01-0,04)	n.s.	0,03(0,02-0,06)	0,02 (0,01-0,04)	n.s.	0,03 (0,01-0,06)	0,02 (0,01-0,04)	n.s.	0,03 (0,01-0,04)	0,02 (0,01-0,04)	n.s.
CRP [mg/dl]	0,4 (0,4-0,5)	0,5 (0,4-0,9)	n.s.	0,6 (0,4-1,3)	0,4 (0,4-0,8)	n.s.	0,6 (0,4-1,2)	0,4 (0,4-0,7)	n.s.	0,6 (0,4-1,0)	0,4 (0,4-0,5)	n.s.
CREA [mg/dl]	1,1 (0,9-1,1)	1,0 (0,9-1,1)	n.s.	1 (0,9-1,2)	1 (0,9-1,2)	n.s.	1 (0,8-1,1)	1,1 (0,9-1,2)	n.s.	1 (0,8-1,2)	1,1 (0,9-1,2)	n.s.

**TOAST**: atherothrombotic	2 (17)	46 (19)	n.s.	4 (10)	44 (20)	<0.0001	15 (17)	33 (20)	0.015	23 (16)	25 (22)	n.s.
cardioembolic	5 (42)	91 (37)		27 (69)	69 (32)		45 (52)	51 (30)		60 (42)	36 (32)	
lacunar	3 (25)	26 (11)		3 (8)	26 (12)		6 (7)	23 (14)		16 (11)	13 (11)	
other	0	20 (8)		2 (5)	18 (8)		5 (6)	15 (9)		12 (8)	8 (7)	
undetermined	2 (17)	61 (25)		3 (8)	60 (28)		16 (18)	47 (28)		31 (22)	32 (28)	

**ECG & TEE**												
VHF on ECG	4 (33)	68 (28)	n.s.	11 (28)	61 (28)	n.s.	24 (28)	48 (28)	n.s.	42 (30)	30 (26)	n.s.
PQ-time [ms]	160 (151-180)	174 (148-180)	n.s.	160 (142-191)	178 (150-198)	n.s.	166 (145-200)	174 (150-190)	n.s.	174 (146-196)	172 (154-200)	n.s.
QTc time [ms]	435 (422-475)	420 (401-475)	n.s.	420 (402-439)	421 (402-442)	n.s.	420 (399-440)	421 (405-442)	n.s.	420 (400-442)	421 (404-441)	n.s.
Impaired LV function	1 (8)	21 (9)	n.s.	4 (10)	18 (8)	n.s.	9 (10)	13 (8)	n.s.	9 (8)	14 (10)	n.s.

**Complications**												
HRD	2 (17)	22 (9)	n.s.	11 (28)	13 (6)	<0.0001 (OR 6.1; 2.6-14.9)	14 (16)	10 (6)	0.01 (OR 3.0; 1.3-7.1)	14 (10)	10 (9)	n.s.
Infections (total)	7 (58)	98 (40)	n.s.	24 (62)	81 (37)	0.007 (OR 2.7; (1.3-5.4)	46 (53)	58 (34)	0.005 (OR 2.1; 1.3-3.6)	69 (49)	17 (12)	<0.0001 (OR 5.4; 2.9-9.9)
Pneumonia	2 (17)	24 (10)	n.s.	7 (18)	19 (9)	0.09	17 (20)	10 (6)	0.002 (OR 3.9; 1.7-8.7)	20 (14)	6 (5)	0.02 (OR 2.9; 1.2 -7.4)

**Outcome**												
length of stay [d]	13 (10-20)	14 (10-20)	n.s.	12 (9,5-21)	14 (10-17)	n.s.	13 (9,5-18)	14 (10-17)	n.s.	14 (10-18)	13 (9-18)	n.s.
mRS ≥3 at three months	7 (58)	118 (48)	n.s.	25 (64)	100 (46)	0.05 (OR 2.1; 1.0-4.2)	51 (59)	74 (44)	0.03 (OR 1.8; 1.1-3.1)	55 (48)	70 (49)	n.s.
dead at three months	1 (8)	18 (7)	n.s.	4 (10)	15 (7)	n.s.	9 (10)	10 (6)	n.s.	8 (7)	11 (8)	n.s.

aHT - arterial hypertension, COPD - chronic obstructive pulmonary disease, CHF - chronic heart failure, CHD - coronary heart disease, AF - atrial fibrillation, SaO_2 _- oxygen saturation, BP - blood pressure, HR - heart rate, HRD - Heart rhythm disturbance, OR - odds ratio, CI - confidence interval. Ordinal/continuous variables are median (IQR), dichotomous variables are n(%).

Both, mild and severe tachycardia was significantly more frequent in patients with a history of AF or AF on admission ECG than in patients without AF, with a stronger association for severe tachycardia and AF. Tachycardia was associated with older age, higher NIHSS scores on admission, larger infarctions on imaging, history of hypertension, history of AF, higher HR on admission and cardioembolic stroke etiology in single factor analysis. The association was stronger for HR≥120 bpm than for HR≥100 bpm. Tachycardia was associated with poor outcome at three months, and patients with tachycardia more frequently died during their hospital stay and at three months, however, these differences were not significant.

Bradycardia <45 bpm occurred in 12 of 256 patients (5%), and was only associated with HR on admission. All other factors analysed did not significantly differ, probably due to number of cases. Bradycardia <60 bpm occurred in 114 patients (44%). Other than HR on admission, there were no factors associated with this endpoint.

After multiple logistic regression analysis neither tachycardia nor bradycardia was predictive of infectious complications. Infections were predicted by lesion size (OR: 1.6, 1.2-2.1 per category, p = 0.0003) and admission and blood glucose on admission (1.01 per mg/dl, 1.0002-1.012, p = 0.04).

HRDs were predicted by lesion size (OR: 1.5, 1.01-2.2 per category, p = 0.04), tachycardia ≥120 bpm (OR: 3.2, 1.2-8.7) and cardioembolic stroke etiology (OR: 4.0, 1.4-11.1, p = 0.008)

Dependency at three months was predicted by older age (OR: 1.04, 1.02-1.07 per year, p = 0.0002), lesion size (OR: 1.7, 1.3-2.2 per category, p = 0.0001) and elevated blood glucose on admission (OR 1.01, (OR 1.001-1.014, p = 0.02). There were trends for history of stroke (p = 0.055) and infectious complications (p = 0.09) to be associated with bad outcome.

**Figure 1:**
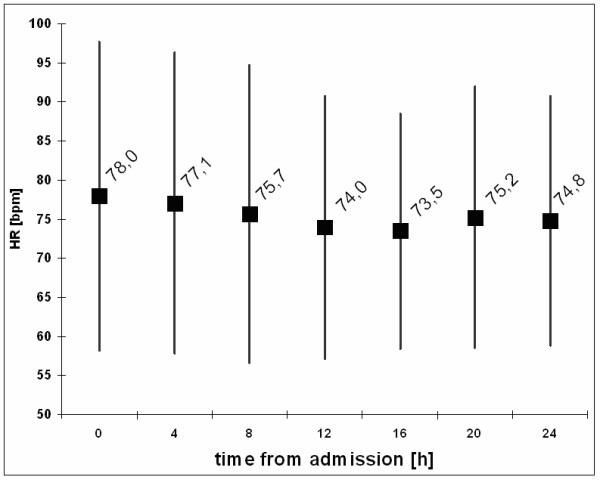
**Mean (± SD) heart rates on admission, at 4, 8, 12, 16, 20 and 24 hours**. Mean values: Large black squares, plus/minus SD: grey lines. There is significant reduction of the mean HR during the first 24 hours after admission.

The course of the mean HR within the first 24 hours is displayed in figure 1.

Mean HR among all patients was highest on admission and sank during the first 24 h. HR dropped by an average of 8 bpm from admission to 12 h, and remained stable from there. The difference between HR on admission, 12 h and 24 hours was highly significant (p < 0.0001).

**Figure 2:**
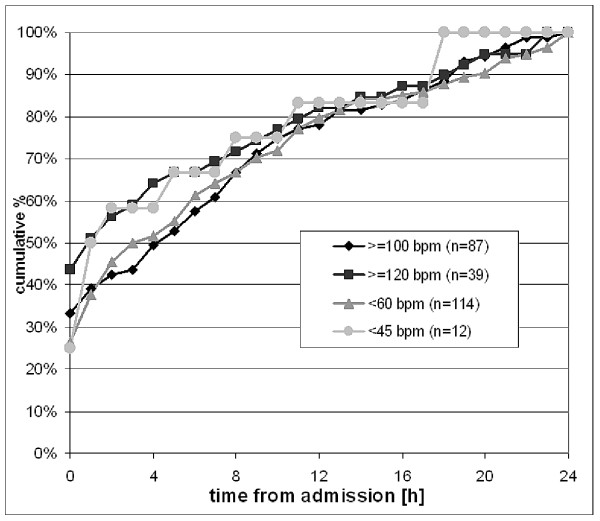
**Cumulative percentage of high and low heart rate violations over the first 24 hours after admission**: Incidence of HR threshold violations over time within the first 24 h after admission. The total number of patients violating the threshold at 24 h is set 100%. Only the first threshold violation was recorded.

Tachycardia according to textbook definition (HR ≥100 bpm) occurred in 87 of 256 patients (34%). More severe tachycardia of HR ≥120 bpm occurred in 39 patients (15%). The onset of both, tachycardia and bradycardia followed a mainly linearly pattern over the first 24 hours after admission; however tachycardia was more frequent on admission compared with bradycardia. Cumulative data on the time of the first detection of tachycardia or bradycardia are given in figure 2.

Severe tachycardia was the rhythm disturbance most frequently present on admission (17 of 39 patients, 33%), followed by mild tachycardia (22 of 87 patients, 33%) and mild bradycardia (30 of 114 patients, 26%). Severe bradycardia occurred with the lowest percentage on admission (3 of 12 patients, 25%).

## Discussion

In our prospective study, significant tachycardia ≥120 bpm occurred in about 10% of acute stroke patients within the first 24 hours after admission. Mainly patients with AF were affected and those already having high HR on admission. Patients with larger infarcts were more likely to exhibit tachycardia. Other authors found cardiac events mainly in patients with large infarctions and high clinical severity [12]. Christensen et al. found tachycardia ≥120 bpm to be related to poor outcome [13] and Steger et al. found admission HR ≥100 bpm to be independently related to mortality [14]. In our study, outcome was worse in patients with tachycardia in single factor analysis. However, these associations did not withstand inclusion of age, lesion size and infectious complications in the final regression model. This could be due to significant interaction between all these factors in acute stroke [15]. We assumed that severe tachycardia would better discriminate between tachycardia with and without prognostic importance, however this may also have led to a case number too small to demonstrate small effects. Patients with cardiac embolism frequently have larger infarctions than other etiologic subgroups. This could to be observed in our data as well, and patients with AF were more likely to develop tachycardia. Thus, inter correlation between tachycardia and lesion size might be so strong that no independent effect of tachycardia on outcome could be seen in our study.

Mean HR was highest on admission and decreased within the next hours, which parallels the course of blood pressure in the first hour after stroke [16], argue for an intense stress reaction of the body leading to sympathoadrenergic responses of the body. This reaction is dependent on lesion size and on psychological stress. The lesion size dependent reaction is in line with other studies reporting stress reactions as causes of troponin increases [17,18] or increases in cardiac events [19]. In the same direction points the observation that higher blood glucose was associated with tachycardia in single factor analysis and that blood glucose was associated with poor outcome. This observation is very much in line with other studies [20].

The occurrence of bradycardia was not predictable by known outcome factors. Only lower HR on admission was associated with bradycardia. On the one hand, bradycardia <45 bpm was too rare in our cohort to reveal robust results. On the other hand, mild tachycardia, which occurred in 44% of all patients failed to be associated with adverse outcome. Bradycardia occurred significantly more frequent during night time than during daytime. Taking the circadian rhythm into account, this result is not surprising [16,21]. Mild tachycardia <60 bpm is not a useful threshold to guide clinical decision making in the setting of acute stroke. In an unselected stroke cohort, bradycardia seems to be mainly a benign frequency reduction in most cases. Life threatening bradycardias are singular events and are poorly predictable.

We did not find a significant preponderance for either brain hemisphere to generate HR disturbances. There was a non-significant trend towards more cardiac complications in left hemispheric lesions which is in contrast to other studies that described preponderance for right hemispheric, especially insular, lesions [13,22,23]. The difference may be explained because in a stroke cohort without pre-selection of lesions of predefined brain regions additional mechanisms such as size of the infarction and individual risk profile cumulatively have a greater role for HR disturbances than specific brain regions. A limitation of our study was that we excluded a significant proportion of patients with severe strokes, namely those, who had to be intubated during the admission process, or for invasive recanalisation procedures, or those, who deteriorated within 24 hours from admission (in total 23 patients, which accounts for almost 10% of the entire cohort). These patients likely would have exhibited a number of additional rhythm disturbances, as we found lesion size and higher NIHSS scores to be associated with tachycardia. Thus, the frequency of arrhythmic periods in a general stroke population was possibly underestimated. On the other side of the spectrum of clinical severity 34 patients with mild strokes had to be excluded as they were referred from the stroke unit in stable conditions before the observation time was completed. This has possibly lead to a slight overestimation of rhythm disturbances in the general stroke population. Overall, the in- and exclusion of patients on both sides of the broad spectrum of clinical severity may have balanced out, and the detected frequency of rhythm disturbances in stroke patients is a good estimate for the frequency in the general stroke population.

## Conclusions

Serious HR threshold violations occur in more than 10% of stroke patients thus justifying intensive monitoring of all stroke patients. Current evidence argues mainly for a significant stress reaction of the body as the reason for HR increases after stroke. Pre-existing cardiac disease further facilitates HR disturbances and cardiac events in this setting. It is thus unlikely that treatment of rate disturbances as such improve prognosis and prevent cardiac events. However, to definitely answer this question, randomised trials including active rate control arms are needed.

## Competing interests

The authors declare that they have no competing interests.

## Authors' contributions

MAR, AR, PUH, JS and DGN designed the study. MAR, AR, RD, DGN collected the data. JS interpreted ECGs, echocardiograms and cardiac events. PUH delivered data on outcome. MAR and EBR drafted the manuscript, all authors revised clinical end points and revised the manuscript. All authors gave final approval to the manuscript.

## Pre-publication history

The pre-publication history for this paper can be accessed here:

http://www.biomedcentral.com/1471-2377/11/47/prepub

## References

[B1] Wolf PA Abbott RD Kannel WB Atrial fibrillation as an independent risk factor for stroke: the Framingham Study Stroke 1991 22 983 988 1866765 10.1161/01.str.22.8.983

[B2] Myers MG Norris JW Hachinski VC Weingert ME Sole MJ Cardiac sequelae of acute stroke Stroke 1982 13 838 842 7147301 10.1161/01.str.13.6.838

[B3] Leys D Ringelstein EB Kaste M Hacke W European Stroke Initiative Executive Committee. The main components of stroke unit care: results of a European expert survey Cerebrovasc Dis 2007 23 344 352 10.1159/000099133 17268165

[B4] European Stroke Organisation (ESO) Executive Committee, ESO Writing Committee Guidelines for management of ischaemic stroke and transient ischaemic attack 2008 Cerebrovasc Dis 2008 25 457 507 18477843 10.1159/000131083

[B5] Adams HP Jr Bendixen BH Kappelle LJ Biller J Love BB Gordon DL Marsh EE Classification of subtype of acute ischemic stroke. Definitions for use in a multicenter clinical trial. TOAST. Trial of Org 10172 in Acute Stroke Treatment Stroke 1993 24 35 41 7678184 10.1161/01.str.24.1.35

[B6] Berger K Weltermann B Kolominsky-Rabas P Meves S Heuschmann P Bohner J Neundorfer B Hense HW Buttner T The reliability of stroke scales. The german version of NIHSS, ESS and Rankin scales Fortschr Neurol Psychiatr 1999 67 81 93 10.1055/s-2007-993985 10093781

[B7] Minnerup J Wersching H Brokinkel B Dziewas R Heuschmann PU Nabavi DG Ringelstein EB Schabitz WR Ritter MA The impact of lesion location and lesion size on poststroke infection frequency J Neurol Neurosurg Psychiatry 2010 81 198 202 10.1136/jnnp.2009.182394 19726403

[B8] Blackburn H Keys A Simonson E Rautaharju P Punsae S The electrocardiogram in population studies. A classification system Circulation 1960 21 1160 1175 13849070 10.1161/01.cir.21.6.1160

[B9] Hamm CW Guidelines: Acute coronary syndrome (ACS). II: Acute coronary syndrome with ST-elevation Z Kardiol 2004 93 324 341 10.1007/s00392-004-0109-x 15085379

[B10] Hamm CW Deutsche Gesellschaft fur Kardiologie- Herz- und Kreislaufforschung. Guidelines: acute coronary syndrome (ACS). 1: ACS without persistent ST segment elevations Z Kardiol 2004 93 72 90 10.1007/s00392-004-1064-2 14740245

[B11] Olsson LG Swedberg K Ducharme A Granger CB Michelson EL McMurray JJ Puu M Yusuf S Pfeffer MA CHARM Investigators. Atrial fibrillation and risk of clinical events in chronic heart failure with and without left ventricular systolic dysfunction: results from the Candesartan in Heart failure-Assessment of Reduction in Mortality and morbidity (CHARM) program J Am Coll Cardiol 2006 47 1997 2004 10.1016/j.jacc.2006.01.060 16697316

[B12] Fink JN Selim MH Kumar S Voetsch B Fong WC Caplan LR Insular cortex infarction in acute middle cerebral artery territory stroke: predictor of stroke severity and vascular lesion Arch Neurol 2005 62 1081 1085 10.1001/archneur.62.7.1081 16009763

[B13] Christensen H Boysen G Christensen AF Johannesen HH Insular lesions, ECG abnormalities, and outcome in acute stroke J Neurol Neurosurg Psychiatry 2005 76 269 271 10.1136/jnnp.2004.037531 15654050 PMC1739524

[B14] Steger C Pratter A Martinek-Bregel M Avanzini M Valentin A Slany J Stollberger C Stroke patients with atrial fibrillation have a worse prognosis than patients without: data from the Austrian Stroke registry Eur Heart J 2004 25 1734 1740 10.1016/j.ehj.2004.06.030 15451152

[B15] Qureshi AI Acute Hypertensive Response in Patients With Stroke: Pathophysiology and Management Circulation 2008 118 176 187 10.1161/CIRCULATIONAHA.107.723874 18606927

[B16] Ritter MA Kimmeyer P Heuschmann PU Dziewas R Dittrich R Nabavi DG Ringelstein EB Blood pressure threshold violations in the first 24 hours after admission for acute stroke: frequency, timing, predictors, and impact on clinical outcome Stroke 2009 40 462 468 10.1161/STROKEAHA.108.521922 19008471

[B17] Ay H Koroshetz WJ Benner T Vangel MG Melinosky C Arsava EM Ayata C Zhu M Schwamm LH Sorensen AG Neuroanatomic correlates of stroke-related myocardial injury Neurology 2006 66 1325 1329 10.1212/01.wnl.0000206077.13705.6d 16525122

[B18] Jensen JK Atar D Mickley H Mechanism of troponin elevations in patients with acute ischemic stroke Am J Cardiol 2007 99 867 870 10.1016/j.amjcard.2006.10.052 17350385

[B19] Prosser J MacGregor L Lees KR Diener HC Hacke W Davis S VISTA Investigators. Predictors of early cardiac morbidity and mortality after ischemic stroke Stroke 2007 38 2295 2302 10.1161/STROKEAHA.106.471813 17569877

[B20] Capes SE Hunt D Malmberg K Pathak P Gerstein HC Stress hyperglycemia and prognosis of stroke in nondiabetic and diabetic patients: a systematic overview Stroke 2001 32 2426 2432 10.1161/hs1001.096194 11588337

[B21] Jehn ML Brotman DJ Appel LJ Racial differences in diurnal blood pressure and heart rate patterns: results from the Dietary Approaches to Stop Hypertension (DASH) trial Arch Intern Med 2008 168 996 1002 10.1001/archinte.168.9.996 18474764

[B22] Abboud H Berroir S Labreuche J Orjuela K Amarenco P GENIC Investigators. Insular involvement in brain infarction increases risk for cardiac arrhythmia and death Ann Neurol 2006 59 691 699 10.1002/ana.20806 16566012

[B23] Colivicchi F Bassi A Santini M Caltagirone C Prognostic implications of right-sided insular damage, cardiac autonomic derangement, and arrhythmias after acute ischemic stroke Stroke 2005 36 1710 1715 10.1161/01.STR.0000173400.19346.bd 16020766

